# Immunity-related GTPase IRGM at the intersection of autophagy, inflammation, and tumorigenesis

**DOI:** 10.1007/s00011-022-01595-x

**Published:** 2022-06-14

**Authors:** Apeksha Bharatgiri Goswami, Dimitrije Karadarević, Natalia Castaño-Rodríguez

**Affiliations:** 1 School of Biotechnology and Biomolecular Sciences, Faculty of Science, UNSW Sydney, Sydney, NSW 2052 Australia; 2 School of Chemical Engineering, Faculty of Engineering, UNSW Sydney, Sydney, NSW 2052 Australia

**Keywords:** IRGM, Autophagy, Xenophagy, Inflammation, Cancer, Immunity

## Abstract

The human immunity-related GTPase M (IRGM) is a GTP-binding protein that regulates selective autophagy including xenophagy and mitophagy. IRGM impacts autophagy by (1) affecting mitochondrial fusion and fission, (2) promoting the co-assembly of ULK1 and Beclin 1, (3) enhancing Beclin 1 interacting partners (AMBRA1, ATG14L1, and UVRAG), (4) interacting with other key proteins (ATG16L1, p62, NOD2, cGAS, TLR3, and RIG-I), and (5) regulating lysosomal biogenesis. IRGM also negatively regulates NLRP3 inflammasome formation and therefore, maturation of the important pro-inflammatory cytokine IL-1β, impacting inflammation and pyroptosis. Ultimately, this affords protection against chronic inflammatory diseases. Importantly, ten *IRGM* polymorphisms (rs4859843, rs4859846, rs4958842, rs4958847, rs1000113, rs10051924, rs10065172, rs11747270, rs13361189, and rs72553867) have been associated with human inflammatory disorders including cancer, which suggests that these genetic variants are functionally relevant to the autophagic and inflammatory responses. The current review contextualizes IRGM, its modulation of autophagy, and inflammation, and emphasizes the role of IRGM as a cross point of immunity and tumorigenesis.

Autophagy is a conserved intracellular process involving the digestion of damaged cytosolic cellular components. The autophagic machinery may be co-opted by the innate immune system for selective digestion of microbial factors, a processed termed xenophagy. Autophagy is regulated by three upstream pathways—the class I PI3K pathway, the AMPK pathway, and the class III PI3K pathway [1]—and the overall process comprises three stages—initiation, maturation, and degradation (Fig. 1). Autophagy is executed by autophagy-related gene (ATG) proteins, which facilitate the process by forming essential complexes with other molecules (Fig. 1).

**Fig. 1:**
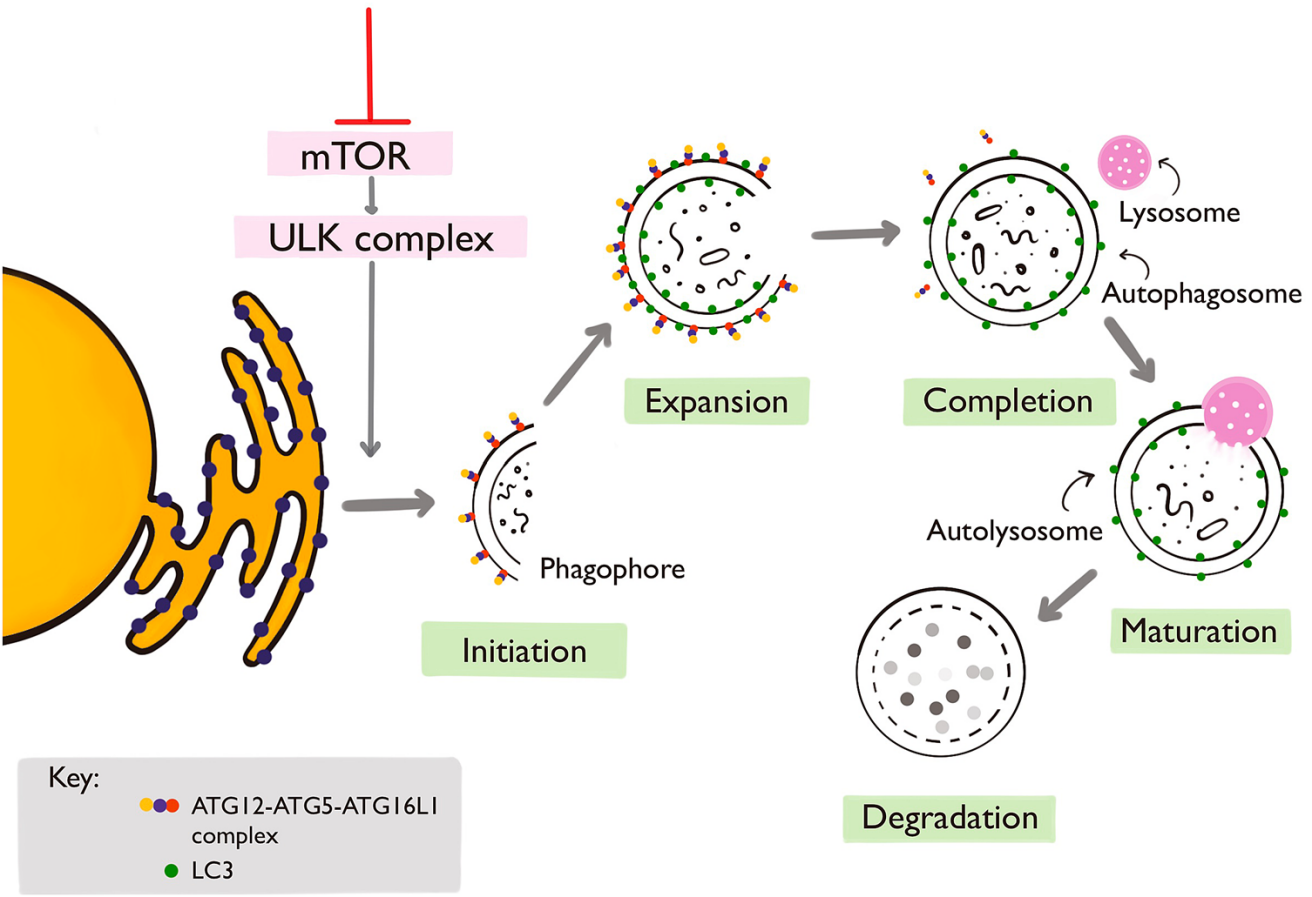
**The autophagy pathway.** mTOR is the fulcrum for autophagic initiation, inhibiting autophagy during cellular metabolic satiety. [1] Inhibition of mTOR triggers autophagy. The activated ULK complex translocates from the cytosol to the endoplasmic reticulum (ER), initiating phagophore formation. [1] The PI3K complex is recruited to the ER and phosphatidylinositol 3-phosphate (PI3P) is generated. [2] PI3P-binding proteins (WIPI1, WIPI2, DFCP1, and Alfy) are recruited to nucleation sites, expanding the phagophore. [2, 3] Phagophore maturation requires two ubiquitin-like conjugation complexes, ATG12-ATG5-ATG16L1 and MAP1LC3 A/B/C-GABARAPs, to form the autophagosome. [1] Specific cargo, such as virulence factors, adhere to the luminal LC3. On completion, the ATG12-ATG5-ATG16L1 complex dissociates from the autophagosome, and this autophagosome fuses with the lysosome to form the autolysosome. [1, 4] The contents are then degraded through lysosomal hydrolases. [4] mTOR, mammalian target of rapamycin; LC3, microtubule-associated protein light chain 3; ULK, Unc-51 like autophagy activating kinase; ULK complex—made of ULK1/2, ATG13, FIP200, and ATG101

## What is IRGM?

The immunity-related-GTPases (IRGs), also known as p47 GTPases, perform a pivotal function in innate immunity. The murine IRG gene family comprises 23 genes as tandem clusters on three chromosomes [5]. Murine models demonstrate that one of these genes, *Irgm1*, influences autophagic flux at the maturation phase when localised to the lysosomal compartment [6]. Murine *Irgm1* modulates autophagy by assisting with autophagosome formation [7] and preventing lysosomal deacidification [7]. *Irgm1* lysosomal localisation is IFN-γ-induced [8] during bacterial infections such as *Salmonella enterica* serovar *typhimurium* infection [9].

Interestingly, this family is reduced to only three genes in humans. This is possibly the consequence of host–pathogen coevolution driven by competition between IRG resistance proteins and pathogen virulence factors as it has been suggested by seminal studies conducted on *Chlamydia muridarum* [10] and *C. trachomatis* [11].

The three identifiable IRG genes in humans are *IRGC*, *IRGQ,* and *IRGM*. *IRGC* and *IRGQ* are located in chromosome 19 and are not involved in human immunity [5]. *IRGM,* which is located in chromosome 5q33.1, is the mammalian ortholog of murine *Irgm1* and has a role in immunity, providing protection against intracellular pathogens [12]. Bekpen et al. [13] identified the process of ancestral *Irgm1* pseudogenation and subsequent reactivation via insertion of the endogenous retroviral element 9 (EVR9) in human lineages. Some murine *Irgm1* autophagy-related functions are performed similarly by IRGM. Of note, IRGM, in association with ATG8, translocates Stx17, an SNARE component, to the autophagosome for lysosomal fusion [14]. In addition, human IRGM functions upstream of autophagic initiation and throughout the autophagic process. Importantly, human IRGM is not IFN-γ-dependant, lacking a γ-activated sequence (GAS) [5]; however, recent evidence suggests it does act as a master negative regulator of cellular interferon responses [13]. There are four *IRGM* isoforms (IRGMa, IRGMb, IRGMc, and IRGMd) with distinct functions. Isoforms IRGMa and IRGMc lack the C-terminal G5 (SAK) motif, which is present in IRGMb and IRGMd [12]. IRGMd isoform becomes embedded within the mitochondrial membrane via cardiolipin, depolarising the membrane, inducing Bax–Bak-dependant cell death by increasing mitochondrial fission [12, 15]. Isoforms IRGMa and IRGMc also exert the same effect at high concentrations, albeit with different kinetic profiles [15]. Tian et al. [16] demonstrated that concentrations of IRGMb, IRGMc, and IRGMd were higher in cancerous tissue compared to paracancerous tissue in melanoma patients, while IRGMa concentrations were relatively lower. In addition, IRGMb was shown to increase melanoma cell survival via increasing autophagic flux, in an HMGB1-dependant manner [16]. This study also established IRGMb as a notable independent risk factor for melanoma progression [16]. Given that generation of alternatively spliced isoforms is frequently associated with drug resistance in cancer therapy, further studies are required to determine the role of IRGM isoforms in diverse tumours.

## IRGM in autophagy

**Fig. 2:**
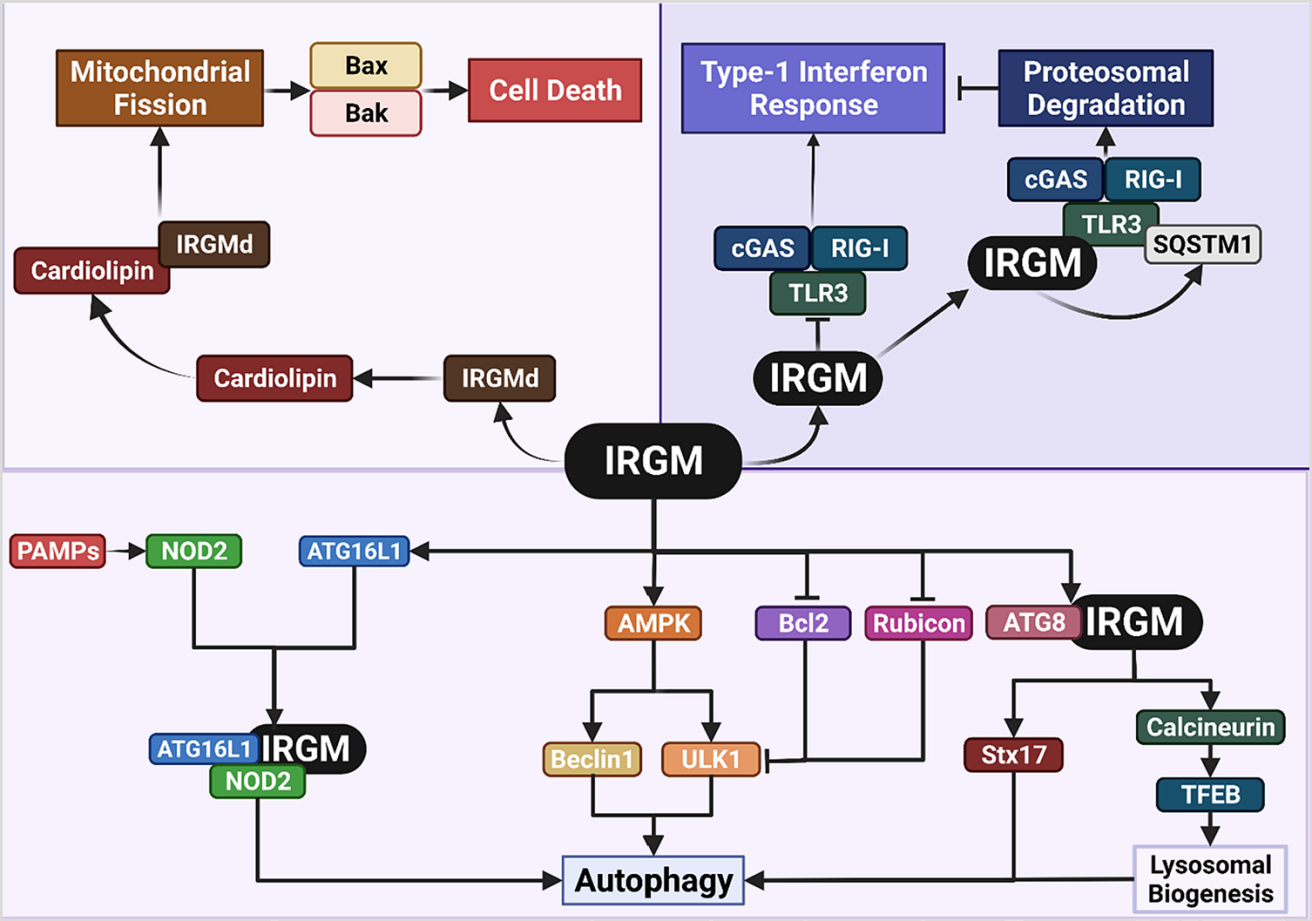
**IRGM regulates autophagy.** IRGM has been shown to be a potent autophagy regulator via five mechanisms: (1) pattern recognition receptors, including NOD2, are triggered upon bacterial infection via PAMPs. Activated NOD2 enhances IRGM binding to ATG16L1, to form a tripartite complex that induces autophagy. (2) IRGM activates AMPK, which in turn phosphorylates ULK1 and Beclin 1 to induce autophagy. (3) IRGM influences the composition of the Beclin 1 complex, by competing with the negative regulators Bcl2 and Rubicon, to trigger autophagy. (4) By binding to ATG8, IRGM induces Stx17 recruitment and stimulates autophagosome-lysosome fusion. (5) Finally, it induces TFEB translocation and lysosomal biogenesis by interacting with calcineurin. Additionally, IRGM isoforms mediate mitochondrial fission by facilitating mitochondrial depolarisation via cardiolipin, potentiating cell death. Furthermore, IRGM prevents type-1 interferon response by sequestering nucleic acid-sensing PRR and inducing their proteasomal degradation via SQSTM1-associated polyubiquitination. PAMPs, pathogen-associated molecular patterns; NOD2, nucleotide-binding oligomerization domain-containing protein 2; AMPK, 5’ AMP activated protein kinase, ATG16L1, autophagy-related gene 16-like 1; ULK1, unc-51 like autophagy activating kinase 1; Bcl2, B-cell lymphoma 2, ATG8, autophagy-related gene 8; Stx17, syntaxin 17; TFEB, transcription factor EB; Bax, Bcl2 associated X; Bak, Bcl2 homologous antagonist killer; TLR3, toll-like receptor 3; cGAS, cyclic GMP-AMP synthase; RIG-I, retinoic acid-inducible gene 1

IRGM modulates selective autophagy including xenophagy and mitophagy, both directly and indirectly. IRGM affects autophagy indirectly through mitochondrial fusion and fission, by modulating mitochondrial membrane collapse [15]. IRGM localisation to the mitochondrial membrane via cardiolipin facilitates mitophagy [15, 17]. IRGM negatively regulates mitofilin stability during mitochondrial depolarisation, resulting in PINK1-Parkin-dependant ubiquitination and subsequent clearance of faulty mitochondria [17]. IRGM also stabilises AMPK in its Thr-172 phosphorylated form [18], which is required for AMPK activation and further induction of autophagy via phosphorylation of the ULK1 complex and Beclin 1 (Fig. 2) [19, 20].

Chauhan et al. [18] demonstrated that IRGM also physically interacts with essential autophagy proteins including ULK1, Beclin 1, ATG14L, and ATG16L1. During microbial insult, IRGM interacts with the pattern recognition receptor (PRR) nucleotide-binding oligomerization domain protein 2 (NOD2) in response to pathogen-associated molecular patterns (PAMPs). NOD2 enhances the K63-linked polyubiquitination of IRGM, which helps IRGM interact with the core autophagic protein ATG16L1, and thus, induces a xenophagic response (Fig. 2) [18]. IRGM also links p62/SQSTM1 to nucleic acid-sensing PPRs including cyclic GMP–AMP synthase (cGAS), Toll-like receptor (TLR) 3, and retinoic acid-inducible gene (RIG-I), enhancing their polyubiquitination and culminating in their proteasomal degradation [21]. Furthermore, IRGM was shown to determine the composition of the Beclin 1 complex, by enhancing the Beclin 1 interacting partners, AMBRA1, ATG14L1, and UVRAG (Fig. 2) [18]. Beclin 1 has two negative regulators, Bcl2 and Rubicon, that bind to its BH3, CCD, and ECD domains. ATG14L1, an enabling regulator, also has a binding site for Beclin 1’s CCD domain. IRGM and ATG14L1 simultaneously bind to Beclin 1 and compete with the negative regulators, thus, initiating autophagy [18]. In addition, absence of IRGM causes proteasomal degradation of the proteins ULK1, ATG14L1, AMBRA1, and ATG16L1, thus indicating a role of IRGM in degradative ubiquitination [22].

Finally, IRGM, in concert with mammalian ATG8 proteins, regulate lysosomal biogenesis, a fundamental process for any autophagic pathway. Kumar et al. [23] elucidated that IRGM’s interaction with transcription factor EB (TFEB) at domain of unknown function (DUF) 3371 and calcineurin PPP3CB causes dephosphorylation of TFEB by PPP3CB, resulting in TFEB nuclear translocation and stimulation of lysosomal biogenesis. The study also demonstrated that IRGM inactivates mTOR, the phosphorylating agent of TFEB, under nutrient starvation, and bacterial and viral infection [23].

## IRGM in cancer

The role of autophagy in cancer is context-dependent, acting as both a promoter and suppressor of tumorigenesis [24]. Evidence that mutations in ATG genes are associated with cancer was first provided when a monoallelic deletion in *BECN1*, an essential autophagy gene encoding Beclin 1, was shown to result in tumorigenesis in breast cancer [25]. This was later observed in 40–75% cases of breast, ovary, and prostate cancers [26, 27]. Studies on other important autophagy genes (*ATG2B*, *ATG3, ATG5, ATG9, ATG12,* and *ATG16L1*) have also been conducted and suggest that autophagy plays a key role in tumorigenesis [28, 29].

IRGM appears to be a fulcrum between immunity and tumorigenesis. Studies on melanoma [16, 30], hepatocellular carcinoma [31], glioma [32], and gastric cancer [33] have shown that IRGM can promote carcinogenesis. In human glioma cell lines, overexpression of IRGM was linked to increased cell colony formation, cell proliferation, and Akt activation [32]. Xu et al. [34] also established that highly expressed IRGM in glioma cells promoted IL-8 production and M2 macrophage polarization via p62/TRAF6/NK-κB signalling. In gastric cancer, mRNA and protein levels of IRGM were shown to be significantly upregulated in the peripheral blood of cancer patients compared to healthy controls, and these levels were higher in stage IV than in stage I cancer patients [33]. Furthermore, there were significant differences in IRGM expression in patients presenting with melanoma, wherein the highest expression occurred in metastatic tumours, followed by primary tumours, and finally, benign adjacent nevus tissue [16]. A similar pattern was observed according to disease stage, where patients in stages III–IV of melanoma showed significantly higher expression of IRGM mRNA and protein levels, compared to patients in stages I and II [16]. In hepatocellular carcinoma (HCC), overexpression of the metallocarboxypeptidase AGBL2, an independent prognostic biomarker that promotes HCC cell survival and proliferation, enhanced autophagy and inhibited apoptosis via IRGM up-regulation [31].

Given the plethora of IRGM-related mechanisms that impact autophagy, a pathway that plays a complex role in cancer per se, it is pivotal to establish the precise mechanisms by which IRGM can promote carcinogenesis.

## IRGM in other inflammatory conditions

**Fig. 3:**
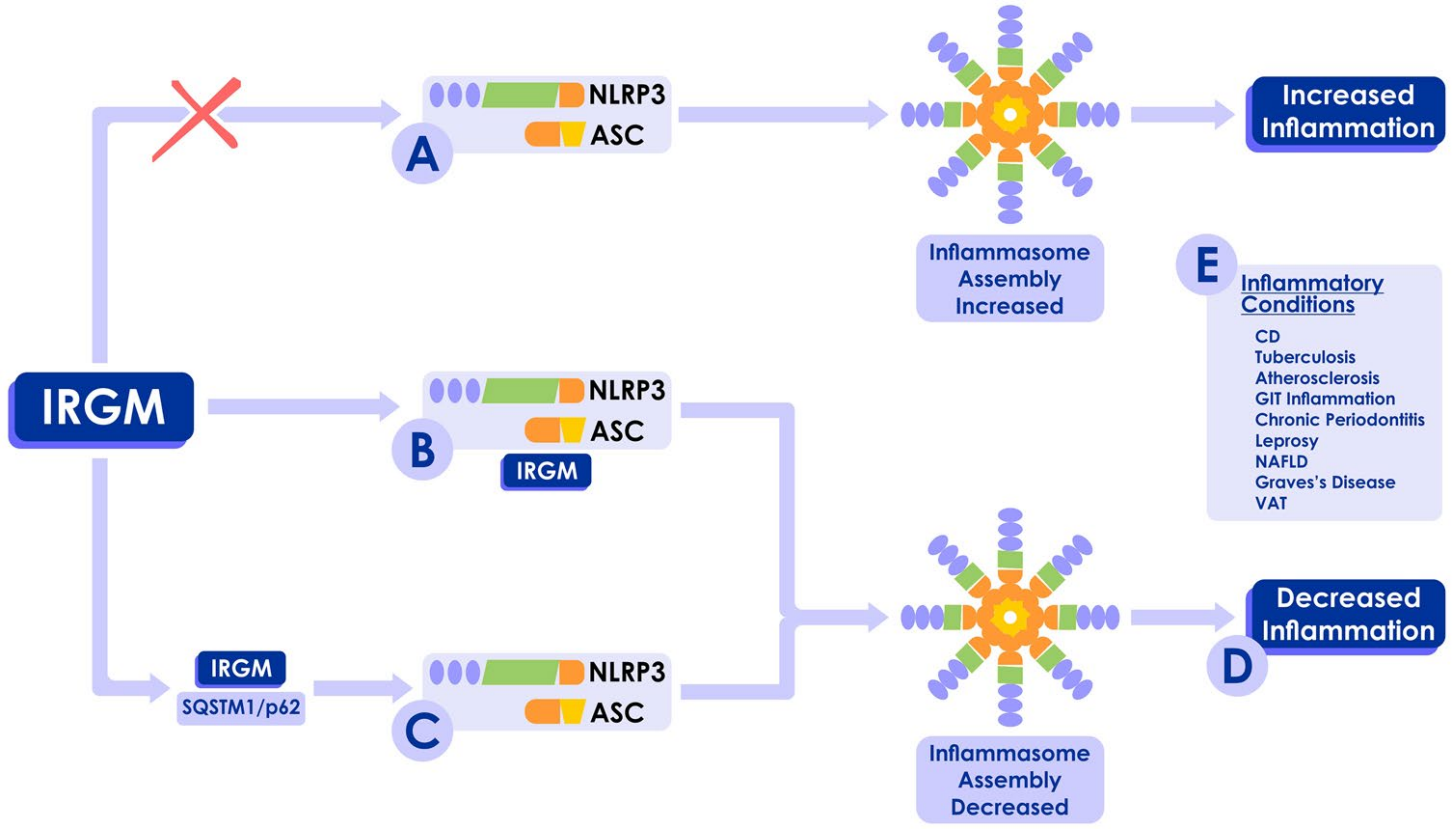
**IRGM and inflammation.** In inflammatory diseases, IRGM controls inflammation by negatively regulating the NLRP3 inflammasome. **A** Upon sensing-specific stimuli, NLRP3 and ASC oligomerize to form a caspase-1 activating scaffold. **B** IRGM physically complexes with NLRP3 inflammasome components and obstructs inflammasome activity. **C** IRGM interacts with SQSTM/p62 and mediates p62-dependent selective autophagy of NLRP3 and ASC and, thus, restricts inflammasome activity. **D** Thus, IRGM suppresses inflammation and provides protection against inflammatory diseases. **E** Inflammatory conditions that have been associated with IRGM. NLR, NOD-like receptors; ASC, apoptosis-associated speck-like protein containing CARD; CD, Crohn’s disease; GIT, gastrointestinal; NAFLD, non-alcoholic fatty liver disease; VAT, visceral adipose tissue. Adapted from Mehto et al. [36].

Inflammation is a hallmark of cancer. In fact, many chronic inflammatory disorders including inflammatory bowel diseases (IBD) (e.g., Crohn’s disease (CD) and ulcerative colitis) [35] and autoimmune diseases (e.g., primary Sjogren syndrome, rheumatoid arthritis, systemic lupus erythematosus, and systemic sclerosis) [36] are well known to significantly increase the risk of cancer. Recent studies demonstrate that IRGM modulates anti-inflammatory processes, particularly in the context of IBD [37]. *Irgm1* deficient murine models have exhibited functional abnormalities in intestinal Paneth cells and hyper-inflammation in colon and ileum, upon dextran sodium sulphate exposure [38]. Mehto et al. [39] have revealed that IRGM is a negative regulator of the NLRP3 inflammasome, suppressing inflammation and providing protection against inflammatory diseases including CD. IRGM has been shown to control inflammation by interacting with SQSTM1/p62 and mediating p62-dependent selective autophagy of NLRP3 and ASC [39]. In addition, IRGM appears to block NLPR3 and ASC oligomerization, hindering inflammasome assembly [39]. Thus, IRGM restricts inflammasome activity and protects from pyroptosis (Fig. 3) [39]. Furthermore, by inhibiting inflammasome activation, *Irgm1* has been shown to negatively regulate cellular inflammation in immune and intestinal epithelial cells in a CD murine model [39]. Knockdown of *IRGM* has resulted in morphology changes of dendritic cells, leading to hyperstability of the immunologic synapse as well as increased T-cell activation [37]. This mechanism might explain the loss of immune tolerance in the intestine and increased adaptive immunity in CD patients who carry *ATG16L1* and *IRGM* risk alleles [40].

Recently, Jena et al*.* [21] demonstrated that IRGM prevents translocation of IFN-α/β transcription factors IRF3 and IRF7 via proteasomal degradation of nucleic acid-sensing PRRs and mitophagy of hyperpolarised mitochondria. Hyperpolarised mitochondria yield mtROS and cause mtDNA soiling, which leads to RIG-I, TLR3, and cGAS activation and concludes with IRF3/7 phosphorylation and nuclear translocation [21]. Furthermore, in vivo* Irgm1*^−/−^ murine models present with mitochondria-dependant type-1 interferonopathy that is tissue specific [41]. An aberrant type-1 IFN response results in increased apoptosis and contributes to human autoimmune disease, such as systemic lupus erythematosus [21, 41]. Indeed, *Irgm1*-null murine models exhibit IFN-1-dependant autoimmune syndrome, which is comparable to Sjogren’s syndrome via defective mtDNA clearance [42]. Thus, by modulating mitochondrial fission [15, 41] and polyubiquitination of PRRs, IRGM prevents type-1 interferonopathy [21]. Advancing our knowledge of IRGM as a master switch of type-1 IFN responses will be beneficial for generating therapeutics against autoimmune disorders.

However, IRGM has also been shown to support immunopathogenesis. In mouse and human intestinal epithelial cell lines, it was found that IRGM can modulate necroptosis and release damage associated molecular patterns to induce gastrointestinal inflammation [43]. Furthermore, Fang et al*.* [44] have demonstrated that *Irgm1* deficient murine models lead to macrophage apoptosis rescue by preventing ROS accumulation and phosphorylation of JNK/p38/ERK in the MAPK pathway. *Irgm1* imparts a pro-inflammatory M1 macrophage phenotype by stabilising M1-associated transcription factors *Irf5* and *Irf8* [45]. In addition, *Irgm1*-haplodeficient mice demonstrated reduced *iNOS* activity in M1 macrophages and reduced M1 polarization [45].

## IRGM during microbial insult

Infectious diseases represent the third leading cause of cancer worldwide. In fact, 15.4% of cancers are attributable to carcinogenic infections [46]. *Helicobacter pylori*, high-risk human papillomavirus (HPV), hepatitis B virus (HBV), and hepatitis C virus (HCV) account for 90% of infection-related cancers worldwide [46].

Human IRGM has been extensively investigated as a xenophagy inducer during bacterial infection [12, 47, 48]. *H. pylori*-infected patients harbouring *IRGM* rs13361189 demonstrated a remarkably increased risk of gastric cancer development [49]. Considering that *H. pylori* induces IRGM downregulation in a strain-dependant manner, *IRGM* polymorphisms and microbial suppression act synergistically to prevent xenophagic clearance [49]. The deletion or knock-down of the murine ortholog *Irgm1* results in increased susceptibility to both intracellular and extracellular bacteria, including *Citrobacter rodentium* [50], *S. typhimurium* [9], *Mycobacterium tuberculosis* [12], *Listeria monocytogenes* [51], and *C. trachomatis* [52]. Numerous mechanisms for increased susceptibility in murine *Irgm1*^*−/−*^ models have been proposed: failure of monocyte maturation upon lamina propia infiltration and apoptosis [50], abolishment of macrophage movement and adhesion [9], and loss of intracellular bacterial restriction mechanisms [52]. In vitro models using *M. leprae* and *M. tuberculosis* corroborate murine models, identifying increased IRGM expression upon infection [12, 53]. Interestingly, impaired *IRGM* expression results in persistent replication of adherent-invasive *Escherichia coli* (AIEC) in epithelial cells and macrophages, which has been implicated in CD pathogenesis, with further increased production of IL-6 and TNF-α [47]. The inability of macrophage-mediated AIEC clearance has been further demonstrated in CD patient-derived macrophages harbouring *IRGM* rs10065172 [54].

IRGM is also a fundamental negative regulator of type-1 IFN response against viral pathogens [21, 55]. IRGM plays a key role in replication of HCV, an important risk factor for hepatocellular carcinoma, by regulating Golgi fragmentation and leading to co-localization of Golgi vesicles with replicating HCV [48]. Furthermore, autophagosome formation is stimulated in HIV-infected and HCV-infected HeLa cells, via IRGM interaction with HIV-NEF and HCV-NS3 [56]. This may aid in viral survival by autophagic degradation of nucleic acid-sensing proteins RIG-I and cGAS [21]. Epithelial and monocytic cells lines with abolished IRGM demonstrate enhanced antiviral properties, including MHC-I presentation and PKR stress granule formation, and are resistant to ZIKV and SARS-CoV-2 infection [55].

While the interaction of IRGM and fungi remains understudied, Rosentul et al*.* [57] investigated the effects of HIV+patient-derived peripheral blood mononuclear cells cytokine stimulation by *Candida albicans* blastoconidia, demonstrating patients harbouring *IRGM* rs13361189 had increased IL-8 levels compared to patients not harbouring this variant. IRGM also plays a pivotal role in limiting parasitic protozoan proliferation, including *Trypanosoma cruzi* and *Toxoplasma Gondii,* by ensuring macrophage maturation and secluding protozoan vacuoles to the lysosome [51, 58].

In the era of the microbiome, the limited number of studies investigating the potential impact of IRGM on whole microbial communities needs to be addressed. This would be particularly pertinent in gastrointestinal disorders. To date, the most relevant study investigating the impact of *IRGM* genetic variants on gut dysbiosis [59] demonstrated that several IBD risk alleles, including *IRGM* rs11741861, are associated with a decreased abundance of the genus *Roseburia* in healthy individuals (FDR = 0.017).

## *IRGM* germline variants associated with disease

*IRGM* polymorphisms have been investigated in relation to cancer including gastric cancer, renal cell carcinoma, and glioma (Table 1) [49, 60–62]. Of these, consistent associations have been found between *IRGM* rs4958847 and rs13361189 and gastric cancer; we [49] showed that rs4958847 decreases the risk of gastric cancer in ethnic Han Chinese populations, while Burada et al. [60] showed comparable results in Caucasian populations. Interestingly, both studies reported borderline associations between *IRGM* rs13361189 and gastric carcinogenesis. *IRGM* rs13361189 is in perfect linkage disequilibrium with a 20-kb deletion located immediately upstream of the *IRGM* promoter gene [63]. This deletion is replaced with seven nucleotides, causing IRGM segregation in the population with two distinct upstream sequences and alters IRGM regulation, which subsequently affects autophagy [63]. Importantly, *IRGM* rs13361189 appears to increase the risk of other types of cancer in Chinese populations, as evidenced by Ge et al. [61] who demonstrated an increased risk of glioma in subjects harbouring this polymorphism.

**Table 1:** Association between *IRGM* polymorphisms and inflammatory conditions including cancer

Meta-analyses				
Disease	Author	Population	Polymorphism	Association
CD	Li et al.[64]	Caucasian	rs13361189	Increases risk
			rs10065172, rs4958847	No association
	Lu et al. [65]	European	rs13361189, rs4958847 and rs10065172	Increases risk
Tuberculosis	Xie et al. [70]	Asian, African, African-American	rs10065172 (only in Asians), rs4958842, rs4859843 and rs4859846	Decreases risk
			rs72553867	No association

Case–control and cohort studies				
Disease	Author	Population	Polymorphism	Association
Ankylosing spondylitis	Xia et al. [77]	Han Chinese, female	rs10065172	No association
			rs4958846	Decreases risk
AITD (Graves’ disease)	Yao et al. [74]	Chinese	rs10065172, rs4958847, and rs13361189	Increases risk
AITD (Hashimoto’s thyroiditis)			rs10065172, rs4958847, and rs13361189	No association
Candidiasis in HIV seropositive patients	Rosentul et al*.* [57]	Tanzanian	rs13361189 and rs4958847	No association
CD	Kee et al. [80]	Malaysian (Malay, Han Chinese, Indian)	rs4958847	No association
			rs11747270 A/G	Increases risk
			rs72553867	No association
	Teimoori-Toolabi et al. [81]	Iranian	rs10065171 and rs4958847	No association
	Pranculienė et al. [82]	Lithuanian	rs4958847	Increases risk
	Na et al. [83]	Korean (Early Onset CD)	rs1000113	Increases risk
			rs72553867	Decreases risk
			rs13361189, rs4958847 and rs10065172	No association
CD fistulising disease	Latiano et al. [67]	Italian	rs4958847	Increases risk
Frequency of CD Ileocolectomy	Sehgal et al. [68]	USA	rs4958847	Increases risk
Complications of postoperative CD Ileocolectomy	Kline et al. [69]	USA	rs13361189	Increases risk
Chronic periodontitis	Folwaczny et al. [76]	German	rs13361189, rs10065172, rs4958847, rs1000113 and rs931058	No association
			rs11747270	Increases risk
Clear cell renal cell carcinoma	Santoni et al*.* [62]	Italian	rs10059011	No association
Gastric cancer	Castaño-Rodríguez et al*.* [49]	Han Chinese	rs4958857	Decreases risk
			rs13361189	Increases risk
Gastric cancer	Burada et al*.* [60]	Romanian	rs4958857	Increases risk
			rs13361189	Decreases risk
Glioma	Ge et al*.* [61]	NA	rs13361189	Increases risk
			rs10065172	No association
Hepatic steatosis	Simon et al. [73]	Caucasian (Framingham Heart Study participants)	rs13361189	No association
Leprosy	Yang et al. [75]	Han Chinese	rs13361189	Increases risk
			rs4958842	No association
Mortality due to severe sepsis	Kimura et al. [79]	Japanese	rs10065172	Increases risk
NAFLD	Lin et al. [84]	Han Chinese (pediatric patients; 6–18 years old)	rs13361189, rs9637876, rs72553867, rs1000113, and rs11747270	No association
			rs10065172	Increases risk
Tuberculosis	Lu et al. [85]	Chinese	rs10065172, rs10051924 and rs13361189	Decreases risk
	Yuan et al. [86]	Han Chinese, Hubei Province	rs4958846	Decreases risk
			rs4958842 and rs4958843	No association
	Song et al. [87]	Korean	rs10065172	Decreases risk
			rs72553867 and rs12654043	No association
	Bahari et al. [88]	Iranian	rs4958843 and rs4958846	Decreases risk
			rs4958842	No association
	King et al. [89]	African-American	rs10065172	Increases risk
			rs11747270	Increases risk
UC	Palomino-Morales et al. [78]	Spanish	rs13361189 and rs4958847	Increases risk
VAT and NAFLD in patients presenting with CD	Simon et al. [72]	Prospective registry in inflammatory bowel Disease study at Massachusetts general hospital (PRISM) cohort	rs13361189 and rs4958847	Increases risk

*CD* Crohn’s disease; *UC* ulcerative colitis; *NAFLD* non-alcoholic fatty liver disease; *AITD* autoimmune thyroid disease; *VAT* visceral adipose tissue; *HIV* human immunodeficiency virus; *NA* not available

Several studies establishing links between *IRGM* polymorphisms and other human inflammatory diseases have been conducted (Table 1) The most established associations have been with CD and tuberculosis. An early meta-analysis by Li et al. [64], including 5183 CD patients and 5571 healthy controls, showed a significant association between rs13361189 and CD, but not rs4958847 and rs10065172. However, a meta-analysis by Lu et al. [65], comprising a much larger study sample size (20590 IBD cases and 27670 controls), has demonstrated that these three *IRGM* polymorphisms (rs13361189, rs4958847, and rs10065172) significantly increase the risk of CD. In addition, subgroup analyses by ethnicity conducted in both meta-analyses [64, 65] revealed significant associations between these *IRGM* polymorphisms and an increased risk of CD among Caucasians but not among Asian populations. Recently, Ajayi et al*.* [66] demonstrated that subjects harbouring *IRGM* polymorphisms (rs13361189 and rs10065172) present with reduced IRGM expression in their serum and terminal ileum, indicating that these disease-associated SNP also affect IRGM expression, not only protein activity. Importantly, *IRGM* polymorphisms also appear to exacerbate symptoms (rs4958847) [67] and complicate prognoses (rs4958847 and rs13361189) [68, 69] following treatment of CD.

A meta-analysis by Xie et al. [70], investigating the association between *IRGM* polymorphisms status and the risk of tuberculosis, comprising 3780 patients with tuberculosis and 4835 controls, reported a decreased risk of this disease in the presence of *IRGM* rs10065172, rs4958842, rs4859843, and rs4859846. The capacity for *IRGM* polymorphisms to affect tuberculosis development appears to be species-dependant, as the variant rs9637876 (−261TT) results in significant protection from *M. tuberculosis*, but not *M. africanum* or *M. bovis* [71].

Other inflammatory diseases that have been associated with *IRGM* polymorphisms include non-alcohol fatty liver disease (rs4958847, rs13361189 and rs10065172) [72, 73], visceral adipose tissue (rs4958847 and rs13361189) [72], autoimmune thyroid disorders (rs10065172, rs4958847, and rs13361189) [74], leprosy (rs13361189) [75], chronic periodontitis (rs11747270) [76], ankylosing spondylitis (rs4958846) [77], ulcerative colitis (rs13361189 and rs4958847) [78], and sepsis (rs10065172) [79].

## Conclusions

Over the past decade, autophagy has evolved from a purely homeostatic tool to a complex axis of immunological, inflammatory, and carcinogenic processes, spurred by several seminal studies. In healthy tissue, autophagy can function as a survival mechanism, maintaining viability during stress by sequestering damaged organelles or intracellular pathogens. IRGM*,* being the only molecule that has been shown to regulate autophagy upon infection, thus, becomes greatly important. IRGM modulates autophagy by promoting the co-assembly of ULK1 and Beclin 1, and by its interaction with key proteins such as ATG16L1, NOD2, and p62. IRGM also affects mitochondrial fusion and fission, regulates lysosomal biogenesis, and restricts NLRP3 inflammasome activity. Consistently, *IRGM* polymorphisms have been associated with human diseases including cancer (gastric cancer, renal cell carcinoma, and glioma), infection (tuberculosis and leprosy), autoimmunity (autoimmune thyroid disorders) and inflammatory disorders (CD, non-alcohol fatty liver disease, and chronic periodontitis). Given the intersection that occurs among autophagy, inflammation, and tumorigenesis, understanding the functional relevance of IRGM could facilitate the translation of new therapies for cancer and other prevalent inflammatory disorders.
